# Work–family conflict, overwork and mental health of female employees in China

**DOI:** 10.3389/fpubh.2025.1483746

**Published:** 2025-04-02

**Authors:** Jun Ma, Laixi Xu, Xuehe Zhang

**Affiliations:** ^1^School of Humanities, Shandong Management University, Jinan, China; ^2^Anhui Kecheng Intelligent Health Co., Ltd, Hefei, China; ^3^School of Public Affairs, University of Science and Technology of China, Hefei, China

**Keywords:** work–family conflict, overwork, mental health, career woman, moderated mediation model

## Abstract

**Introduction:**

The “Green Paper on the Mental Well-being of Chinese Career Women” indicates that around 85% of Career Women face mental health challenges such as anxiety, depression, and anger, with these issues being more common than in their male counterparts in China. Both work and family are identified as two major contributors to these problems. Utilizing Conservation of Resources theory, this paper examines work–family conflict and overwork as significant explanatory variables and develops a moderated mediation model to investigate the mechanisms affecting mental health issues among Chinese career women.

**Methods:**

Data were gathered through a questionnaire survey, with 500 questionnaires distributed and 393 responses received. Hayes’ PROCESS macro for SPSS was employed to examine moderated mediation models, with Bootstrap resampling set at 1000.

**Results:**

(1) Work–family conflict (abbreviated as WFC) is significantly associated with emotional exhaustion (abbreviated as EE) and mental health problems in career women, with emotional exhaustion serving as a mediator between work–family conflict and mental health. (2) Overwork is positively linked to emotional exhaustion and influences the relationship between work–family conflict and emotional exhaustion. (3) Overwork also moderates the mediation effect of work–family conflict on mental health via emotional exhaustion, amplifying the mediation effect when career women are overburdened.

**Discussion:**

This study provides fresh insights into the mechanisms underlying mental health issues among career women, offering valuable information for addressing these challenges.

## 1 Introduction

### 1.1 Background

According to labor market data of World Bank 2023, the female labor force participation rate in China reached 60.54%, meaning nearly two-thirds of eligible Chinese women are engaged in labor and work. This proportion exceeds the average of high-income countries globally (54%) (1). Furthermore, with China’s population aging intensifying, there is considerable pressure on social labor resources. Against this dual backdrop, the health and vitality of Chinese female labor force are particularly crucial for the socio-economic development of China.

The 2019 “Green Paper on the Mental Well-being of Chinese Career Women” revealed numerous health challenges faced by Chinese women in the workplace. Most career women self-assess their health as fair or poor, with anxiety, depression, and social isolation increasingly becoming their major concerns. Approximately 85% of career women experienced mental health problems in the past year, with about one-third feeling anxious or depressed from time to time, and 7% reporting constant anxiety or depression. The Green Paper also indicated that around 90% of career women experienced negative emotions and psychological symptoms in the past 3 months. Nearly half of them reported feeling irritable, anxious, confused, or scared during this time. These issues were more common among women than men in the Chinese workforce (2).

Based on the above discussion, the mental health of Chinese career women is currently in an unfavorable state. However, understanding the origins of these issues is a pressing topic for further research. Existing literature suggests that among the various influencing factors, work and family are the primary contributors to women’s mental health issues (3, 4). Despite a clearer understanding of the major precursors affecting mental health, the mechanism how work and family interactions impacts the mental well-being of female employees remain unclear, presenting a significant research gap. Noted above, we conducted interviews with Chinese career women, revealing that WFC and overwork play crucial roles in their mental health. Yet, how these factors lead to mental health issues remains to be explored.

### 1.2 Literature review and conceptual model

In China, women have traditionally been the primary caregivers within families. However, with a significant increase of females entering the labor market, the traditional male-breadwinner model has undergone fundamental changes. This shift requires women to balance work and home duties, inevitably resulting in work-family and role conflicts (3, 5). WFC can be defined as “An inter-role conflict in which the role pressures from the work domain are, to some extent, incompatible with the family domain” (6). According to the Resource Conservation Theory, the Scarcity Hypothesis posits that work and home duties compete for an individual’s limited resources. When individuals attempt to fulfill both roles simultaneously, resource scarcity causes role conflict, exacerbating conflict between work and family domains (7, 8). Existing research indicates that WFC can detrimentally affect the mental issues of the staffs, with a greater impact on women compared to men (9–11).

Furthermore, data from the “China Labor Statistical Yearbook 2021” shows that in 2020, urban employed individuals in China had an average weekly working time of 47.0 h. In comparison, OECD countries averaged 36.7 h per week in 2019. This disparity underscores that Chinese workers have significantly longer work hours compared to the OECD average (12). Some sectors in China even experience the “996” phenomenon, where employed individuals toil from 9 AM to 9 PM, 6 days a week, highlighting prevalent issues of overwork in the country (13, 14). “Overwork” is a state where workers accumulate fatigue due to prolonged and intense work activities. Prior literature has revealed that long working hours could pose health risks to employees, and for women, the mental health issues caused by overwork are particularly pronounced compared to men (15).

Based on the literature review above, both WFC and overwork significantly affect the mental health of women in workplace. However, the specific mechanisms through which these factors deteriorate women’s mental health remain unclear. Therefore, this study aims to construct a model to elucidate the mechanisms of WFC and overwork on the mental health of women in workplace, thus addressing gaps in previous research. In accordance with conservation of resources theory, both WFC and overwork exhaust an individual’s psychological resources, leading to emotional exhaustion (16). Emotional exhaustion refers to a chronic state of being physically and emotionally overextended, exhausted and drained by excessive job demands and continuous work hassles (17). This exhaustion undermines an individual’s self-regulation abilities, triggering a cascade of mental and behavioral responses such as anxiety, depression, anger, avoidance, aggression, and non-cooperation (18). Previous studies have shown that EE is a precursor to mental health. When EE accumulates to a certain extent and is not eliminated, it can arouse people’s mental health problems (19).

Throughout this process, due to identity and role cognition reasons, most career women aspire to achieve balance between their home and work duties, making WFC particularly prone to arise (20). When conflicts occur and cannot be resolved, women in the workplace experience chronic stress. Prolonged stress inevitably depletes limited psychological resources, leading to EE and subsequent mental health issues (7, 21, 22).

As previously mentioned, overwork is a common phenomenon in China and can lead to emotional exhaustion in individuals (23). In addition to its main effects, overwork can further exacerbate the emotional exhaustion caused by WFC among working women, thereby acting as a moderating factor. In other words, the strength of the relationship between WFC and emotional exhaustion varies depending on the degree of overwork. When working women have longer working hours, they expend more psychological resources, resulting in fewer resources available to manage WFC, which in turn intensifies emotional exhaustion (24).

**Figure 1:**
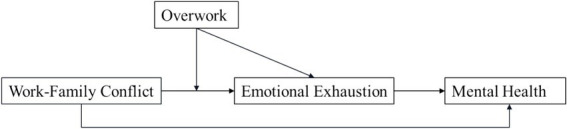
The conceptual model.

Based on this analysis, we have developed a moderated mediation model to investigate the explanatory mechanism linking WFC and overwork to EE and mental health issues in women. This study proposes that WFC leads to EE, which in turn affects the mental health of career women —thus EE mediates the relationship between WFC and mental health. Simultaneously, overwork moderates the relationship between WFC and EE. Combining these factors, overwork not only moderates the relationship between WFC and EE but also exerts a moderating effect on the relationship between WFC mediated by EE and mental health, thereby forming a moderated mediating effect. Figure 1 describes the conceptual model proposed in this research.

In summary, the main problem explored in this paper is how the intersection of work and family impacts the mental health of career women. We argue that work–family conflict and overwork are significant factors that negatively affect their mental health. These challenges drain their psychological resources, leading to emotional exhaustion and ultimately harming their overall mental well-being.

## 2 Data and methods

### 2.1 Procedure and subjects

Data in the reperch were collected via questionnaire survey. The questionnaire consisted of three parts: an informed consent form, demographic variables, and measures. All measures were administered using a 7-point Likert scale. To mitigate common method bias, participants’ average weekly work hours were obtained from the human resources departments of their respective companies. The work identification numbers of participants were included in the questionnaire design to match respondents’ work hours with their responses.

To ensure the scientific and representative nature of the sample, participants were drawn from three industries in Shandong and Anhui province of China. Industry types included service, manufacturing, and IT sectors. These industries account for most female employees in China, thereby ensuring the representativeness of the sample.

Generally, a sample size of 300 or ten times the number of items is required to ensure statistical robustness. A total of 500 questionnaires were distributed, with 393 valid responses collected. Ages of participants range between 18 and 55 years (mean age = 37.37 years). The average weekly work hours were 49.9.

### 2.2 Constructs and instruments

#### 2.2.1 Work–family conflict

The measurement items of WFC were adopted from Grzywacz and Marks (24). The scale includes 8 items: 4 for WFC and 4 for family–work conflict. Cronbach’s *α* value for WFC was 0.929.

#### 2.2.2 Emotional exhaustion

The scale, comprising six items, was adopted from the Oldenburg Burnout Inventory, which has demonstrated good validity (17, 25). Cronbach’s α coefficient for EE was 0.925.

#### 2.2.3 Overwork

Based on prior research, working exceeding 50 h per week is considered overwork (26). In this research, overwork was coded as 0 and 1, where 0 represents weekly work hours less than 50 h, and 1 represents weekly work hours greater than 50 (overwork).

#### 2.2.4 Mental health

Conceptualizations of mental health vary widely, including dimensions such as satisfaction, happiness, and depression (27). To reduce respondent burden and enhance measurement validity globally, single-item indicators have become increasingly popular (28). In this research, single-item was used to assess mental health: “How do you rate your mental health at the present time?” Responses ranged from 1 (Excellent), 2 (Very Good), 3 (Good), 4 (Fair), 5 (Poor), 6 (Very Poor) to 7 (Extremely poor) (29).

#### 2.2.5 Control variables

Base on the prior studies, we control the age, education level (1. below high school, 2. high school, 3. bachelor, 4. master, 5. doctorate), marital status (1 for married,0 for unmarried), and workplace type (1 for indoor office, 2 for business premises, 3 for workshop, 4 for outdoor).

### 2.3 Data analysis

Data analysis employed Hayes’ (30) PROCESS to examine moderated mediating models (Module 7, first stage moderation), with Bootstrap resampling set at 1000. Hayes’ PROCESS can simultaneously calculate moderation and mediation effects, and provide direct effects, indirect effects, and confidence intervals of Bootstrap, making it a commonly used tool for analyzing moderation mediation models. The analysis proceeded in three steps: first, testing the mediating effects among WFC, EE, and mental health; second, examining the moderating effect of overwork on EE; third, testing the moderated mediation effect of overwork on the WFC via EE to mental health.

## 3 Results

Before formal data analysis, we conducted descriptive statistics on variables, including mean, SD, and correlations in Table 1. WFC positively correlated with mental health and EE, with correlation coefficients of 0.471 (*p* < 0.01) and 0.217 (*p* < 0.01), respectively. Overwork showed significant positive correlations with mental health and WFC, with coefficients of 0.434 (*p* < 0.01) and 0.349 (*p* < 0.01), respectively. The average age of participants was 37.37 years. The mean of weekly working hours in the past 6 months were 49.9 h, with 214 respondents working over 50 h (classified as overwork) and 179 working fewer than 50 h.

**Table 1:** Descriptive statistics.

	1	2	3	4	5	6	7	8	Mean	SD
Mental health (1)	1								5.62	1.07
Emotional exhaustion (2)	0.012	1							5.42	1.35
Work–family conflict (3)	0.471^**^	0.217^**^	1						5.93	0.94
Overwork (4)	0.434^**^	0.076	0.349^**^	1					0.54	0.49
Marriage status (5)	0.006	0.149^**^	0.016	−0.067	1				0.71	0.45
Age (6)	−0.022	−0.067	−0.002	0.053	0.448^**^	1			37.37	7.48
Education (7)	0.064	0.096	−0.078	0.047	−0.004	−0.110^*^	1		1.85	1.05
Workplace (8)	−0.106^*^	−0.150^**^	−0.102^*^	−0.035	−0.081	−0.001	0.072	1	1.85	0.73

**p < 0.01 *p < 0.05.

The scale measurements, all based on self-reports from female employees, may raise a common methodology bias problem. The Harman single factor test results showed that the explanation rate of a single factor for all variance was 33.31% among total variance of 70.22%, which did not exceed 40%, and it could be concluded that there was no common method variance problem (CMV).

After the descriptive statistics and common methodology bias test, we proceeded with model testing. Specifically, we employed Baron and Kenny’s procedure to conduct the mediating effects (31). The steps included: (1) testing whether WFC significantly predicts EE, (2) examining whether WFC significantly predicts mental health, (3) assessing whether EE significantly predicts mental health, and (4) testing the combined effects of WFC and EE on mental health if the previous equations hold. The details are shown in Table 2.

**Table 2:** Mediating effect test.

Dependent variables	Emotional exhaustion		Mental health		
Model	M1	M2	M3	M4	M5
Work–family conflict		0.198**		0.472**	0.497**
Emotional exhaustion					0.125**
Marital status	0.141*	0.138*	−0.004	0.003	0.014
Age	0.006	0.003	−0.016	0.008	0.008
Education	0.086	0.072	−0.070	−0.105*	−0.096*
Workplace	−0.133**	−0.114*	−0.111*	0.066	0.080
*R* ^2^	0.049	0.087	0.017	0.236	0.25
Adjusted *R*^2^	0.039	0.076	0.007	0.226	0.239
*F* Value	4.972**	7.4**	1.65	23.913**	21.46**

**p<0.01, *p<0.05.

According to the results from Models 2, 4, and 5 in Table 2, it was found that WFC positively predicts EE (*β* = 0.198, *p* < 0.01) and mental health (*β* = 0.472, *p* < 0.01). When EE was included in the regression predicting mental health, the direct effect of WFC on mental health was not weakened or disappeared (*β* = 0.497, *p* < 0.01), indicating partial mediation. In addition, bootstrap was conducted to calculate the mediating effect value. The indirect effect value of WFC on mental health is 0.024 with BootLLCI and BootULCI [0.0447, 0.058], which indicates mediating effect of EE.

Next, we tested whether overwork moderates the relationship between WFC and EE. After standardizing all variables, a moderation term (overwork × Work–Family Conflict) was created. Sequential regression analyses were conducted: first controlling for all variables predicting EE, then adding WFC, followed by both WFC and overwork, and finally adding the interaction term. The results from Model 4 in Table 3 (*β* = 0.163, *p* < 0.01) confirmed that overwork indeed moderates the effects of WFC on EE.

**Table 3:** Moderating effect test.

Model	M1	M2	M3	M4
Work–family conflict		0.198**	0.255**	0.349**
Overwork			0.160**	0.145**
Overwork × Work family conflict				0.163**
Marital status	0.141*	0.138*	0.117*	0.117*
Age	0.006	0.003	0.017	0.017
Education	0.086	0.072	0.058	0.058
Workplace	−0.133**	−0.114*	−0.116*	−0.116*
*R* ^2^	0.049	0.087	0.109	0.125
Adjusted *R*^2^	0.039	0.076	0.095	0.109
*F* Value	4.972**	7.4**	7.881**	7.882**

**p<0.01, *p<0.05.

**Figure 2:**
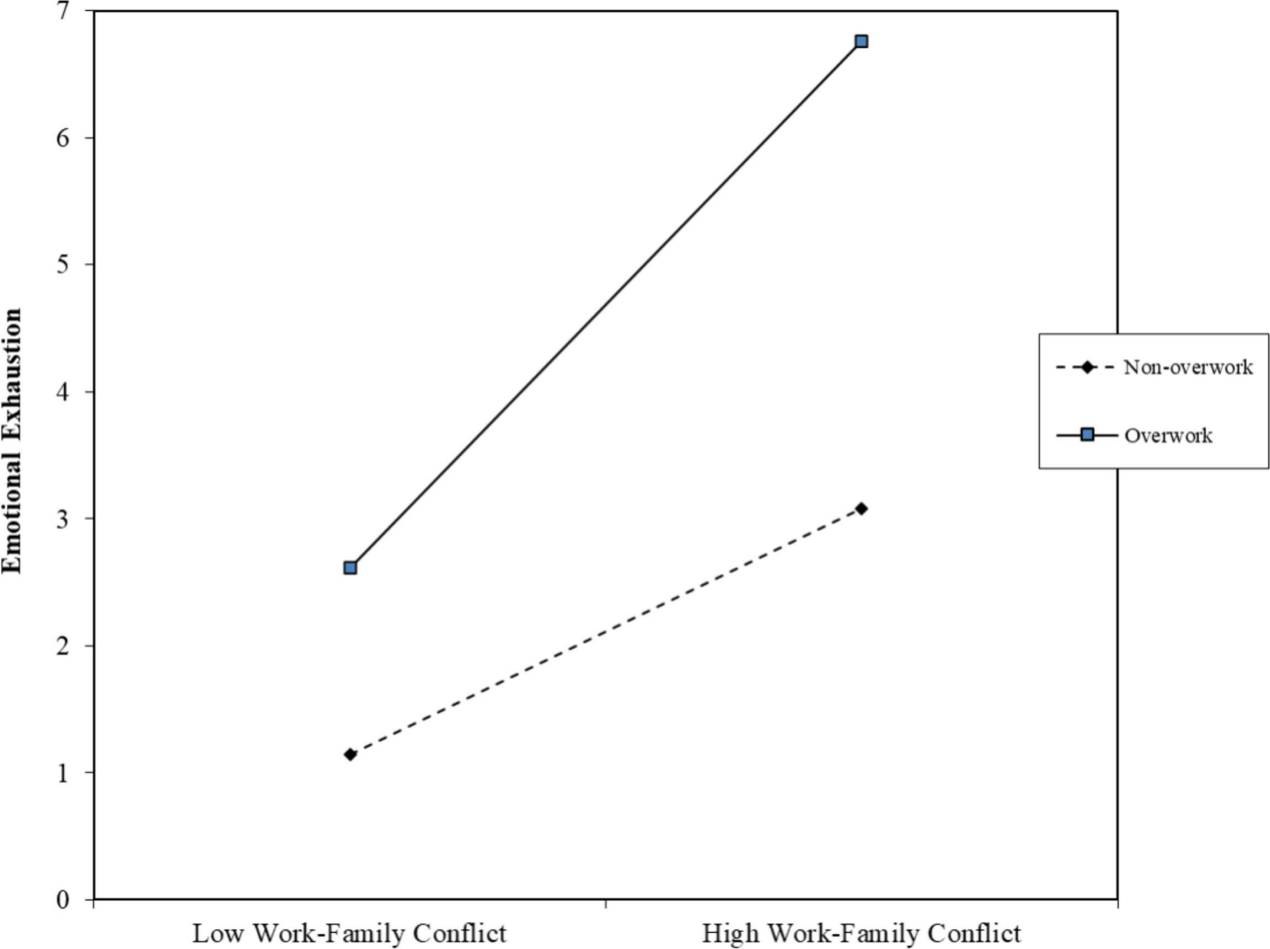
Moderating effect of overwork.

To visually depict the moderating role of overwork, a moderation effects plot was generated using PROCESS v3.5. As shown in Figure 2, regardless of whether overwork is at 0 or 1, there is a significant positive effect. However, the slope when overwork equals 1 (average weekly working hours over 50 h) is notably steeper compared to when it equals 0 (average weekly working hours less than 50 h), indicating that the positive relationship between WFC and EE is more pronounced in the presence of overwork.

Finally, we employed Hayes’ PROCESS to test the overall model’s moderated mediation effects, with Bootstrap resampling set at 1000. Table 4 presents the results of overwork moderating the indirect effects of WFC on mental health via EE. The BootLLCI to BootULCI values (0.0057 to.0832) did not include zero, confirming the presence of moderated mediation effects in the overall model.

**Table 4:** Moderated mediation effect test.

Direct effect					
Effect	SE	*T*	*p*	LLCI	ULCI
0.4967	0.0454	10.9523	0.0000	0.5859	0.4075
Indirect effect					
Overwork	Effect	BootSE	BootLLCI	BootULCI	
0	0.0067	0.0128	0.0172	0.0351	
1	0.0435	0.0177	0.0132	0.0821	
Index of moderated mediation					
	Index	BootSE	BootLLCI	BootULCI	
Overwork	0.0368	0.0201	0.0057	0.0832	

**Figure 3:**
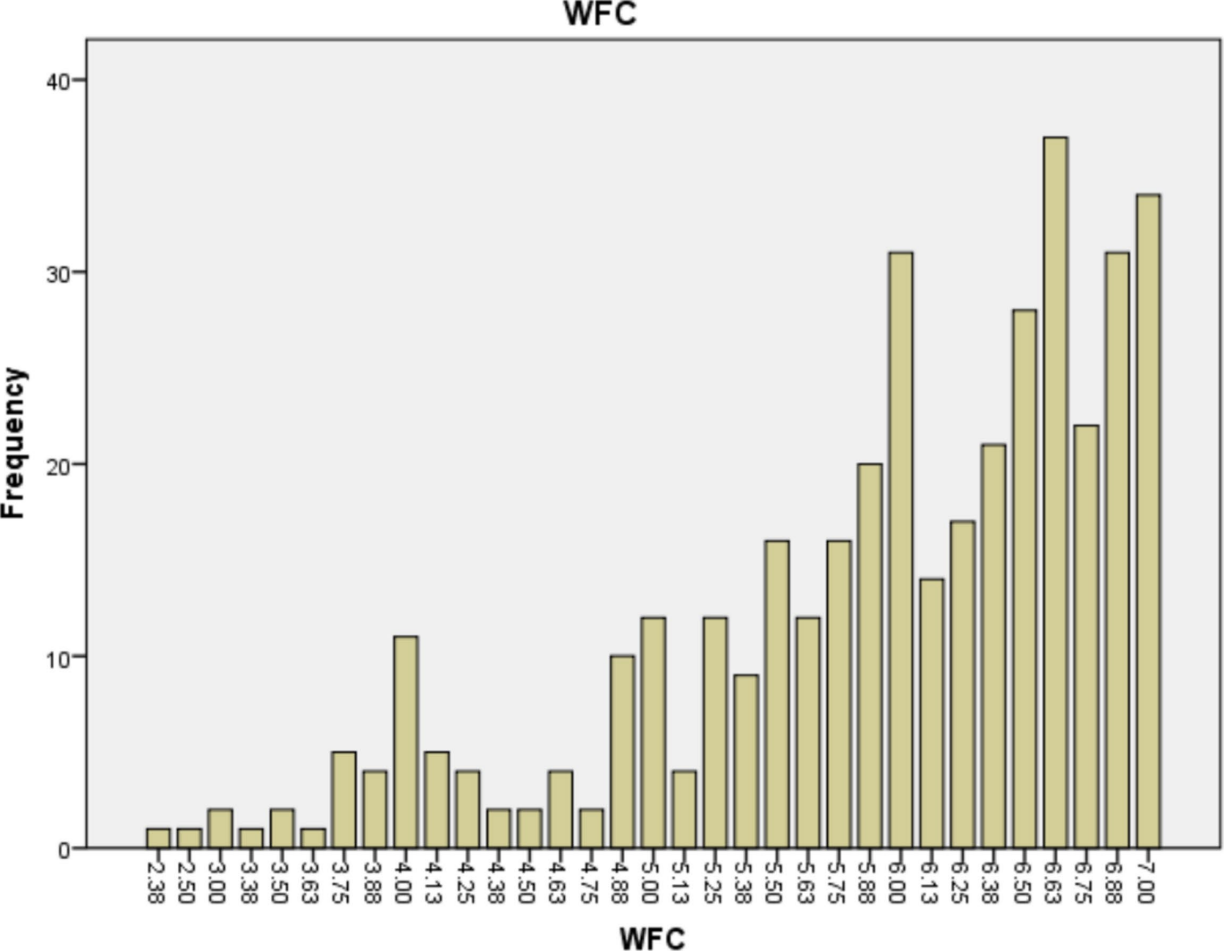
Frequency distribution of WFC.

**Figure 4:**
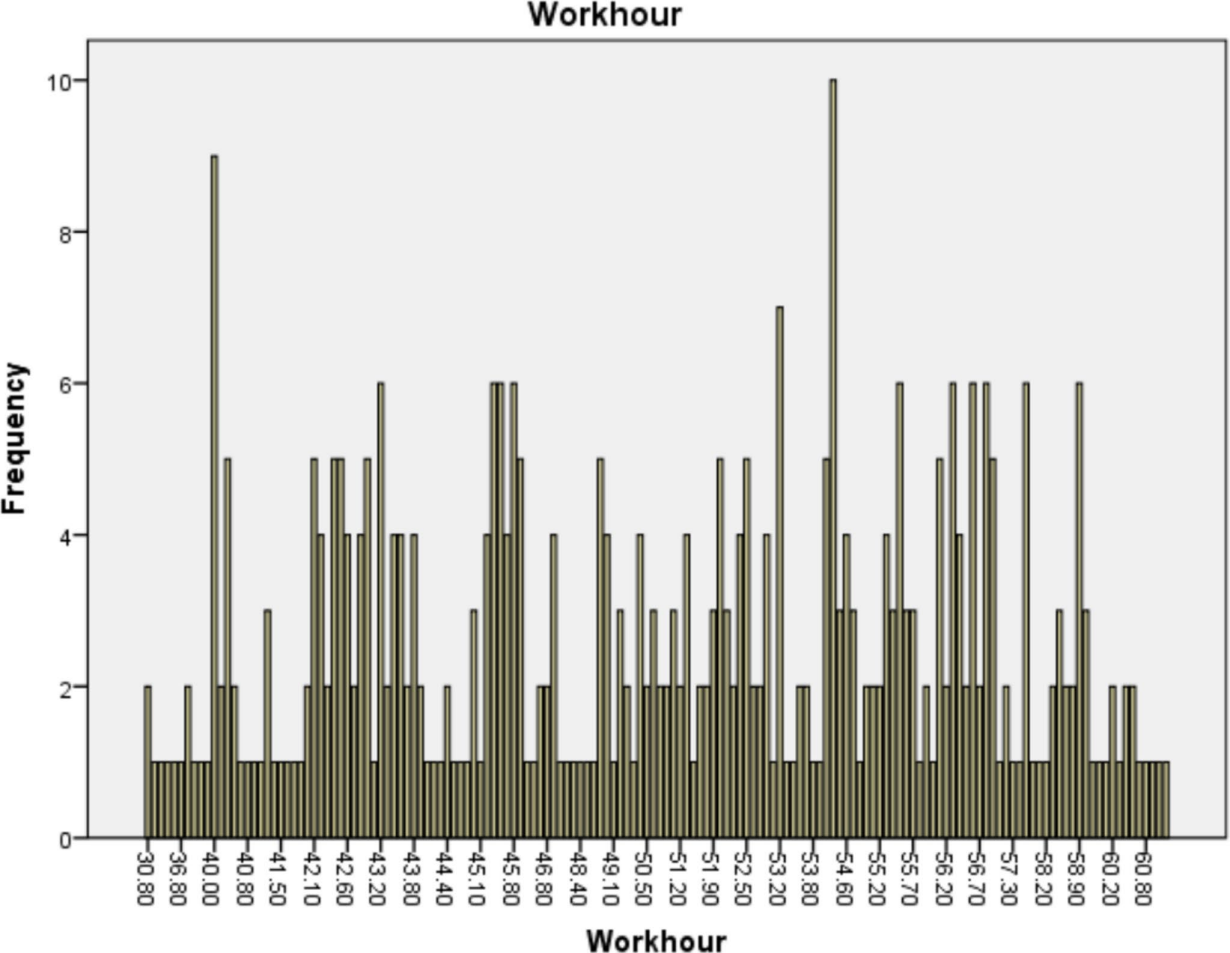
Frequency distribution of working hour.

Finally, in order to comprehensively present the impact of WFC and Overwork on mental health, we calculated the frequency distribution of WFC and average weekly working hours among career women. The specific values are shown in Figures 3, 4.

In conclusion, these findings highlight the complex interplay among WFC, overwork, EE, and Mental Health, underscoring the importance of considering both direct and indirect pathways in understanding their relationships in organizational contexts.

## 4 Discussion

In traditional Chinese culture, women are often seen as the caregivers while men are seen as the earners. Females tend to bear greater responsibilities in the family, particularly in nurturing and educating the next generation. Upon entering the workforce, due to gender role expectations, WFC becomes inevitable (32). Based on our data collection, the mean of WFC among career women is 5.93 (on a scale from 1 to 7), with over 80% experiencing levels above 5, indicating a significantly high prevalence of WFC.

Long-term WFC inevitably leads to role conflicts for women (33). Limited by personal resources such as time and energy, individuals struggle to fulfill incompatible roles and duties from both work and home domains simultaneously. When resources like time and energy are depleted, EE ensues, making it difficult for individuals to effectively cope with various pressures, thereby leading to mental health issues (34, 35). Our empirical research confirms a significant positive correlation between WFC and EE, as well as between EE and mental health, highlighting WFC as a crucial precursor to mental health problems among career women.

Reducing WFC can have positive implications for women’s mental health. When WFC arises, women face pressures from both work and family spheres, making it challenging for individuals to proactively mitigate such conflicts. Therefore, organizations play a key role in reducing WFC (36, 37). While some Chinese organizations have begun implementing policies such as flexible working hours, childcare assistance to meet employee needs, these initiatives require substantial human and financial resources and may not be universally feasible. Cultivating a supportive organizational culture that values family and enhancing managerial support may prove more valuable than formal family support policies. When employees receive affirmation of family values and psychological support from their managers, their psychological resources are replenished, potentially reducing perceptions of work-family role conflict (38). This approach may yield effects comparable to formal family support policies. Hence, future efforts could focus on enhancing informal measures for employees to create a family-friendly work environment. Companies should reconsider their relationship with employees. Additionally, there should be a shift away from pressure-oriented practices toward mutually beneficial solutions for both enterprises and employees.

In the Chinese workplace, another major issue facing career women is overwork. According to the data we have collected, the average weekly working hours for our study participants is as high as 49.9 h, nearing the threshold for overwork. Among 393 individuals surveyed, 214 reported working over 50 h per week, accounting for 54.5%.

Past research has yielded conflicting results regarding the relationship between overwork and health. Some scholars argue that overwork negatively affects both the physical and psychological well-being of employees (39). Conversely, others contend that there is no significant correlation between overwork and health (40). Controlling for numerous covariates and individual heterogeneity, a study in China suggests little evidence that long working hours directly affect workers’ health (41). The inconsistency in these findings may stem from the possibility that the impact of overwork on health is not direct but interacts with other variables (42). Excessive work demands consume a significant amount of an individual’s time, potentially amplifying the negative effects of WFC, leading to increased EE and greater sources of stress for individuals (43, 44). Our research findings indicate that overwork not only correlates positively with EE but also moderates the relationship between WFC and EE, thereby influencing the mental health of female professionals. Through interviews with career women, we identified WFC and overwork as two major concerns they face. Addressing the widespread mental health issues among female professionals requires a comprehensive understanding of how WFC and overwork interact. These issues are inherent to the nature of work and persist as long as women are engaged in the workforce, and cannot be alleviated solely through individual efforts. Based on this analysis, concerted efforts from various sectors of society are needed to prevent the further spread of psychological issues among Chinese career women and to improve their mental health promptly. Organizations and families alike need to focus on mitigating EE through both formal systems and informal psychological support, aiming to reduce chronic stress and thereby enhance the mental well-being of female professionals in the workplace.

Our research findings reveal that Marital Status is positively associated with EE and Mental Health among career women. Married women exhibit higher levels of EE and mental health compared to unmarried women, suggesting that marriage may lead to more WFC, thereby impacting EE and mental health. The workplace shows a negative correlation with EE and mental health among female employees. Females working in workshops and outdoors are more susceptible to EE than those in office or business premises, indicating that job nature significantly influences emotional depletion and consequently impacts mental health.

Furthermore, educational attainment among women in the workforce exhibits a negative correlation with mental health. This may indicate that higher education enhances individuals’ psychological resilience, reducing the occurrence of mental health issues. Alternatively, it might indicate that better-educated women often earn higher incomes, facing less financial stress and, therefore, fewer mental health problems. Hence, it is important to focus on married women in low-income and challenging work environments, as they are particularly vulnerable to mental health challenges.

## 5 Conclusion

This study examines how family and work affect the mental health of career women. While previous literature has established that these factors affect the mental health of career women, the mechanisms through which this occurs have not been fully clarified. We introduce two key variables, WFC and overwork, and employ the moderated mediation model to explain how family and work impact the mental health of females. According to empirical findings, we discovered that WFC significantly depletes psychological resources among career women, leading to EE and ultimately deteriorating mental health. Overwork not only directly contributes to EE but also intensifies the effect of WFC on EE, further exacerbating mental health issues among career women. Within contexts of excessive workload, the indirect impact of WFC on worsening mental health through EE is further intensified, demonstrating a moderated mediation effect.

In summary, this study provides a fresh perspective on the factors affecting mental health among career women by focusing on WFC and overwork. These insights aim to improve our understanding of the mechanisms at play and offer potential solutions for addressing mental health challenges faced by career women.

## Data Availability

The raw data supporting the conclusions of this article will be made available by the authors, without undue reservation.
